# Iatrogenic Inflammatory Fibrosis of Hard Palate in a 13-Year-Old Female Patient

**DOI:** 10.1155/2013/871081

**Published:** 2013-12-17

**Authors:** Shanthan Mettu, Radhika Muppa, G. Siva Prasad Reddy, Srinivas Nallanchakrava, Sri Veda Gurugubelli

**Affiliations:** ^1^Department of Pediatric Dentistry, Panineeya Institute of Dental Sciences, Kamalanagar, Dilsukhnagar, Hyderabad, Andhra Pradesh 500060, India; ^2^Department of Oral and Maxillofacial Surgery, Panineeya Institute of Dental Sciences, Kamalanagar, Dilsukhnagar, Hyderabad, Andhra Pradesh 500060, India

## Abstract

Palatal swellings are rare in children and the incidence differs from that of the adult counterparts. When the palatal swellings do arise in children, they usually are palatal abscess from periapical region, and few cases like pleomorphic adenoma in young adults have also been reported. But inflammatory fibrosis of palate in children is a rare occurrence. Inflammatory fibrosis is formation of excess fibrous connective tissue in an organ or tissue, as a reparative or reactive process. This report describes an unusual case of iatrogenic inflammatory fibrosis on the palate due to extraction of tooth number 22 in a 13-year-old female patient. The patient presented with a single large well-circumscribed oval palatal swelling that was soft, fluctuant, not fixed, and nontender. Surgical excision of the lesion was done and it was sent for histopathological assessment. The biopsy showed fibrous tissue with collagen fibers, spindle shaped fibroblasts, neovascularization, RBCs, chronic inflammatory cells, and traces of salivary gland and nerve tissue.

## 1. Introduction

Rapidly growing, aggressive palatal swellings are relatively rare in children when compared to adults. Among all the palatal swellings, pleomorphic adenoma is the most frequently encountered lesion in young adults [1]. Cases of iatrogenically caused inflammatory fibrosis have not been reported or explored to a large extent in the literature, especially in children. Tooth extraction is one of the most frequent dental procedures performed in a dental office and is sometimes accompanied by intra- and postsurgical complications. Fibrosis is a rare complication after extraction and is characterized by formation of excess fibrous connective tissue in an organ or tissue, which can be reparative, reactive, benign, or pathological [2]. We hereby report a case of a 13-year-old girl with inflammatory fibrosis on the hard palate caused as a complication after extraction of tooth number 22.

## 2. Case Report

A 13-year-old female patient reported to the Department of Pediatric Dentistry, Panineeya Dental College, Hyderabad with a complaint of swelling in the palate region for the last 15 days. When detailed history was obtained, patient gave no history of systemic disease or allergies. Patient presented with congenitally missing tooth number 21. She observed a swelling for which she reported to an undisclosed private dental office where tooth number 22 was extracted. The nodular swelling on the buccal aspect of tooth number 22 disappeared and a swelling on the palatal aspect appeared within two days of extraction and rapidly increased to the present size, for which the patient reported to our institution.

**Figure 1:**
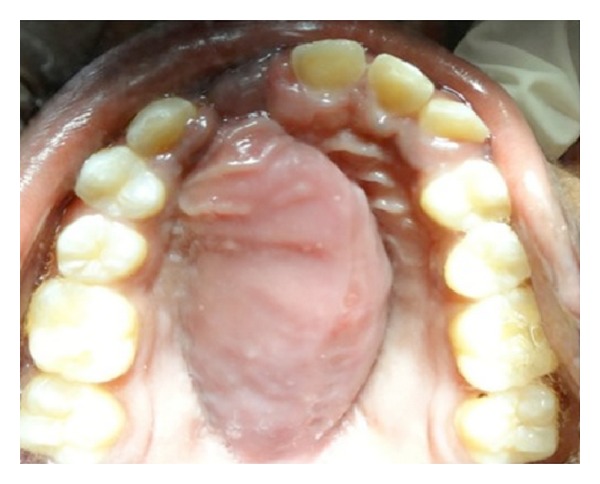
Intraoral preoperative photograph showing palatal swelling.

**Figure 2:**
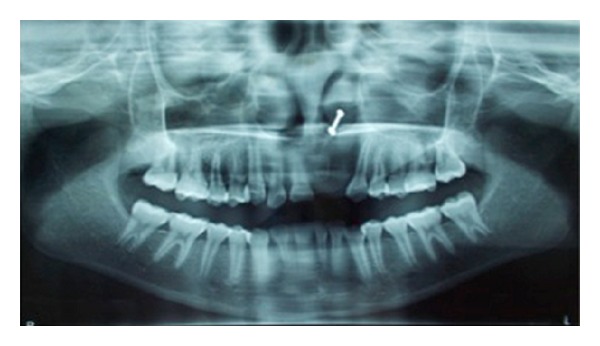
Orthopantomograph showing missing tooth number 21 and tooth number 22.

Patient presented with a 4.5 × 2.7 × 0.8 cm linear swelling, oval in shape, towards the left side extending from the palatal aspect of tooth number 21 to tooth number 26 (Figure 1). The swelling presented as soft, fluctuant, sessile, and nontender. The orthopantomograph and the maxillary occlusal view radiograph revealed a diffuse radiolucency with relation to the extracted tooth number 22 (Figure 2). During the first visit fine needle aspiration cytology (FNAC) was done and the microscopic features showed mixed inflammatory cell infiltrate chiefly lymphocytes and neutrophils within a fibrous background, suggestive of infected cystic fluid. The patient was prescribed broad-spectrum antibiotics for five days and was recalled at regular intervals.

**Figure 3:**
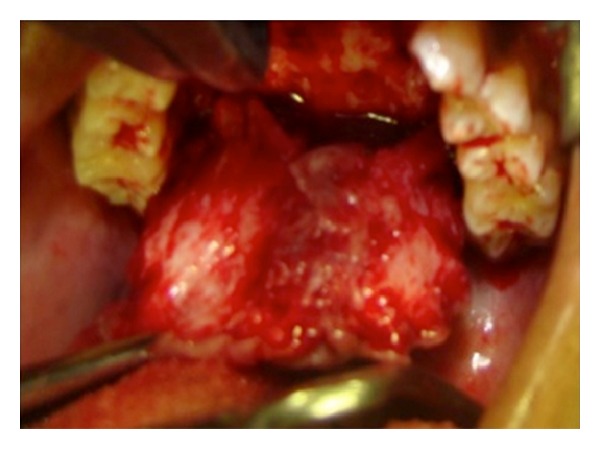
Intraoperative photograph showing excision of the lesion.

**Figure 4:**
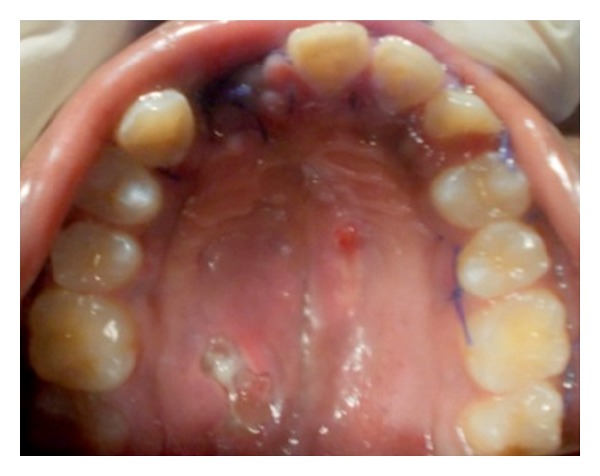
1 week postoperative photograph showing palatal healing.

It was observed that there was no difference in the size of the swelling, even after one week, so enucleation of the lesion was planned under general anaesthesia (GA). Complete haematological tests along with a radiograph of chest PA view were ordered as a preoperative protocol and the case was posted for treatment under GA. Nasotracheal intubation was done and nerve block with 2% lignocaine was given around the lesion. Crevicular incision was performed around the gingival margin on the palatal aspect extending from the regions of 16 to 26, anteroposteriorly. A full thickness mucoperiosteal flap was raised and complete dissection of the lesion was done separating it from the palatal mucosa (Figure 3). Wound closure was done using 3-0 Vicryl and the acrylic feeding plate was placed, which was fabricated prior to the surgery. Extubation of the nasotracheal tube and recovery of patient were uneventful. Patient was recalled after one week for review and subsequently advised to discontinue the acrylic plate after 10 days (Figure 4).

**Figure 5:**
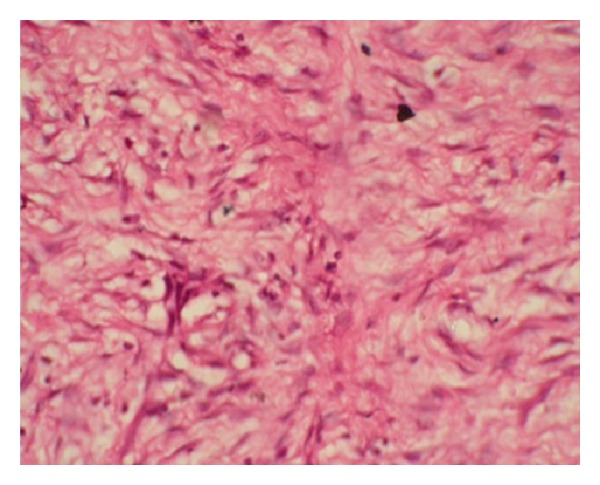
Photomicrograph showing fibrous connective tissue consisting of plump, spindle shaped fibroblasts.

**Figure 6:**
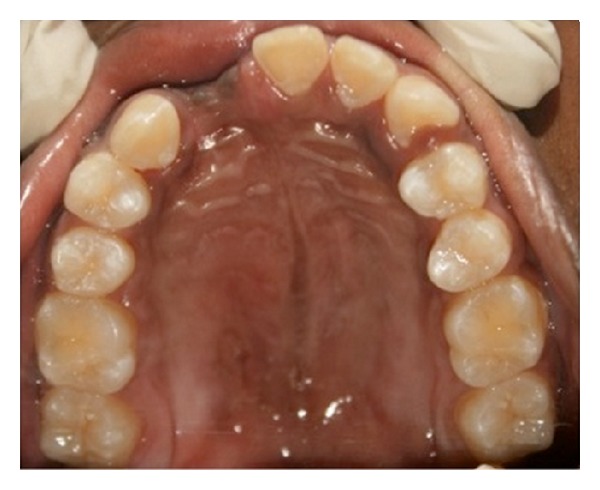
6-month postoperative photograph showing good healing of the palate with no recurrence of the lesion.

The excised specimen was sent for biopsy and microscopy revealed fibrous connective tissue consisting of plump, spindle shaped fibroblasts neovascularization with extravasated red blood cells. It also showed infiltrate of chronic inflammatory cells with no evidence of salivary gland tissue or nerve tissue, thus suggestive of inflammatory fibrosis (Figure 5). The patient has been under followup for the last 6 months and no evidence of reoccurrence appeared (Figure 6).

## 3. Discussion

Damage to the surrounding soft tissues is easily inflicted during negligently performed local anesthetic technique and extraction procedures. In this present case, it could have been any of the two. More serious complications are caused when deeper structures are traumatized when the procedure is done without good knowledge of technique and the anatomy of the tissues. These complications can appear during or after the procedure. The complications may range from infection inflammation trauma to nerves resulting in variable nerve dysfunctions [3].

Inflammatory fibrosis manifests as a postextraction complication caused by possible localized iatrogenic trauma to the soft tissues, deregulating the wound healing process at either the proliferative or remodeling stages. It may also be caused if the irritant persists in the tissues continually during the process of healing. Fibrosis can occur with or without inflammation [4].

Fibrosis is similar to the process of scarring, which involves immigration, proliferation of fibroblasts, and also extracellular matrix deposition by these fibroblasts, which is followed by collagen synthesis. Researchers have suggested that NLRP3 inflammasome is a key cellular sensor that results in generation of an inflammatory response. This inflammasome mediates the activation and recruitment of inflammatory cells to the site of injury as an initial response. Many observations suggest that tissue remodeling and fibrosis are similar, but a delicate alteration in the biosynthetic pathway of reconstruction of extracellular matrix may affect the balance [5].

Complications resulting from rapid palatal injection of local anesthetic solutions, particularly those containing a vasoconstrictor, have been reported, which include ischemia, allergic reactions, epithelial desquamations, traumatic injuries, ulcerations, fibrosis, and also necrosis [6].

Here in the present case, the clinical, radiographic, and histological features were correlated to arrive at a final diagnosis of iatrogenic inflammatory fibrosis.

## 4. Conclusion

Though a rare occurrence, inflammatory fibrosis of hard palate may result as a complication of tooth extraction due to iatrogenic factors. So a thorough knowledge of the clinical, radiographic, and histological features of fibrosis is essential for a proper diagnosis. In spite of the fact that the complications are sometimes inevitable, comprehensive knowledge about the technique and anatomy is necessary for preventing iatrogenic challenges during tooth extraction.
